# Effect of a helium and oxygen mixture on physiological parameters of rats with cerebral arterial air embolism

**DOI:** 10.3389/fphys.2024.1388331

**Published:** 2024-05-13

**Authors:** V. A. Palikov, N. B. Pavlov, R. R. Amirov, A. M. Ismailova, N. A. Borozdina, Yu. A. Palikova, I. A. Dyachenko, O. N. Khokhlova, T. I. Ponomareva, V. A. Rykov, A. T. Logunov, A. N. Murashev, V. M. Baranov

**Affiliations:** ^1^ Institute of Biomedical Problems of the Russian Academy of Sciences, Moscow, Russia; ^2^ Branch of the Shemyakin-Ovchinnikov Institute of Bioorganic Chemistry, Russian Academy of Sciences, Pushchino, Russia; ^3^ Closed Joint Stock Company «Specialized Design Bureau of Experimental Equipment at the Institute of Medical and Biological Problems of the Russian Academy of Sciences», Khimki, Russia

**Keywords:** helium and oxygen mixture, heliox, cerebral arterial air embolism, ischemic stroke, medical oxygen, SD rats

## Abstract

**Introduction:** Cerebral arterial air embolism (CAE) is a serious and potentially dangerous condition that can interrupt the blood supply to the brain and cause stroke. One of the promising gas mixtures for emergency treatment of air embolism is an oxygen-helium mixture.

**Methods:** We modeled CAE in awake rats by injecting air into the common carotid artery. Immediately after CAE, animals were either untreated or underwent hyperbaria, oxygen inhalation, heated air inhalation, or helium-oxygen mixture inhalation. Body temperature, locomotor activity, respiratory and cardiovascular parameters were monitored in the animals before CAE modeling, and 3 and 24 h after CAE modeling.

**Results:** After 3 hours of CAE modeling in awake rats, depression of the nervous, cardiovascular and respiratory systems, as well as decreased body temperature were observed. 24 h after CAE modeling multifocal cerebral ischemia was observed. Normobaric helium-oxygen mixture inhalation, on par with hyperbaric treatment, restored body temperature, locomotor activity, respiratory volume, respiratory rate, and blood pressure 3 hours after CAE, and prevented the formation of ischemic brain damage lesions 24 h after CAE.

**Discussion:** Thus, inhalation of a heated oxygen-helium gas mixture (O2 30% and He 70%) immediately after CAE improves the physiological condition of the animals and prevents the foci of ischemic brain damage formation.

## 1 Introduction

An intravascular air embolism is an air bubble that obstructs the flow of blood in arteries and/or veins. Historical cases of air embolism date back to 1769 when the Italian anatomist Giovanni Morgagni discussed cerebral arterial air embolism (CAE) (Fries et al., 1957). Arterial air embolism can be caused by trauma, such as pulmonary barotrauma or thoracic wounds. Iatrogenic vascular air embolism is a rare but serious condition that can lead to significant morbidity and mortality (Kerrigan and Cooper, 2022).

Air entry into the carotid and coronary arteries can result in immediate stroke or cardiovascular symptoms. Cerebrovascular occlusions caused by air embolism require prompt emergency treatment and reperfusion (Wang et al., 2021).

Therapy for intravascular air embolism, whether venous or arterial, involves 100% oxygen inhalation. The use of oxygen under normobaric conditions is an emergency therapy for decompression syndrome and hypoxia (Wendling, 1993; Longphre et al., 2007). Breathing 100% oxygen during emergency treatment in environments where hyperbaric chambers are not available is the most important and effective therapy because oxygen not only dissolves bubbles but also compensates for hypoxia in the affected tissues (Köhler et al., 2022). This treatment can increase the oxygen content of the blood and improve tissue oxygenation, potentially reducing the risk of complications and improving prognosis. It has been noted that oxygen therapy halts the penumbra region and allows reperfusion to begin at later times. Several studies have demonstrated that the use of 100% oxygen reduces ischemic lesion volume (Flynn and Auer, 2002; Singhal et al., 2005; Henninger et al., 2007; Michalski et al., 2011; Wang et al., 2023). These findings suggest that oxygenation under normobaric conditions could be employed prehospital or upon arrival to extend and enhance penumbral survival in patients with ischemic stroke. The use of 100% oxygen is one of the possible therapies under normobaric conditions.

Air generated as an embolus can be a potential therapeutic target (Aboudara et al., 2014). It can be assumed that hyperbaric therapy itself reduces the size of gas bubbles in blood vessels by following Boyle’s Law, which states that the surface area and volume of a gas bubble are inversely proportional to pressure. However, hyperbaric oxygen therapy (HBOT) has proven to be more effective in endogenous or exogenous gas embolism (Fakkert et al., 2023; Marsh et al., 2023). HBOT is the most common emergency therapy for embolism. HBOT not only reduces the size of the bubbles, but may also help prevent cerebral edema by reducing the permeability of blood vessels while maintaining the integrity of the blood-brain barrier (Muth and Shank, 2000; Marsh et al., 2023). It also diminishes the adherence of leukocytes to damaged endothelium (Muth and Shank, 2000). Oxygen under hyperbaric conditions can increase tissue oxygenation. Although CNS oxygen toxicity is a rare occurrence, it can manifest as an episode of oxygen toxicity accompanied by seizures during HBOT (Heyboer et al., 2017).

The use of gas mixtures against the background of air embolism affects circulatory parameters and the partial pressure of the gases in the vessels. The diffusion capacity and low density of helium allow it to improve oxygen transport through the membrane of the alveolar-capillary complex (Sergysels et al., 1978; Verstappen et al., 1978; Ryan et al., 2023). It is important to note that helium-oxygen mixture (heliox) is used in a heated state. The specific heat capacity of gaseous helium at 20°C and normal atmospheric pressure is 5296 J/(kg·K) and that of air is 1005 J/(kg·K). In order to avoid any possible negative effects of helium on the body in the form of cooling, which can occur due to the high specific heat capacity of helium, heliox is heated. The heated gas mixture of 70% helium and 30% oxygen has a positive effect on pulmonary gas exchange (Liet et al., 2015; Beurskens et al., 2013; Zhang et al., 2014).

In addition to improving respiratory function, studies have shown that inhalation of heliox can have a positive effect on ischemic organs. Evidence suggests that heliox can reduce infarct volume in animal models of focal cerebral ischemia-reperfusion (Pan et al., 2007) and cell damage in *vitro* models of brain injury (Coburn et al., 2008). The effectiveness of a gas mixture containing helium and oxygen has been demonstrated in a model of cardiac ischemia/reperfusion in rabbits (Hale et al., 2014), preventing necrosis and infarct volume. The effectiveness of a helium-oxygen mixture (75% He, 25% O2) has been demonstrated in a model of cerebral ischemia/reperfusion in rats with cerebral artery occlusion, which was demonstrated on tests of motor deficit and in the analysis of brain lesion area (David et al., 2009).

It is possible that a mixture of 30% (28%–32%) oxygen and 70% (68%–72%) helium could impact the progression of CAE due to the presence of both gases. To investigate the potential impact of breathing heliox on gas bubbles in the bloodstream, a model of right-sided intravascular air embolism of the brain was used. This is because ischemia of the brain arteries can cause visual changes in rat behavior and depression of the main vital systems of the organism, including cerebral ischemia.

Taking into account the previously described effects of heliox, we proposed the idea of a possible application of this gas mixture in CAE. Our aim was to investigate the efficacy of heliox in a model of CAE as an emergency prophylactic measure when a person does not have access to a hyperbaric chamber. The only emergency therapy is the inhalation of normobaric oxygen. Therefore, inhalation of 100% oxygen under normobaric conditions was chosen as the standard therapy. We did not consider HBOT because in our case we were mimicking the immediate measures against CAE that occur during the time interval when a person is transported to the hyperbaric chamber. However, we also studied a group of animals using hyperbaric conditions to demonstrate the effect of excessive atmospheric pressure on air embolism.

## 2 Materials and methods

### 2.1 Animals

40 male Sprague Dawley (SD) rats aged 4–5 weeks with Specific-pathogen-free (SPF) status were received from the laboratory of animal breeding center “Puschino” (Scientific production association “Nursery of laboratory animals” of The Branch of the M.M. ShemyakinYu. A. Ovchinnikov Institute of Bioorganic Chemistry of the Russian Academy of Sciences (BIBCh RAS)). Prior to the experiment, the animals were acclimated at least for 14 days. Animals were 8–9 weeks of age at the start of CAE modelling. All procedures were carried out in accordance with the standard operating procedures of the laboratory and were approved by the bioethical commission of the Institutional Animal Care and Use Committee of BIBC RAS (Protocol No 922/22 from 27.12.2022).

During the experiment, animals were kept in the two corridors barrier-type facility with the automatic change of day and night time (08:00-20:00 - “Day”, 20:00-08:00 - “Night”) and the renewal of the room air at least 12 times hourly. Temperature (20°C–24°C) and humidity (45%–65%) were continuously monitored in each animal housing room automatically using a computerized Eksis Visual Lab system (EVL, Practik-NC). Standard Diet for laboratory mice and rats Mucedola 4RF21 autoclavable (Mucedola s. r.l., Via G. Galelei, 4–20019 Settimo Milanese MI, Italy) and filtered tap water (MilliRO, Millipore) were given *ad libitum*.

**FIGURE 1:**
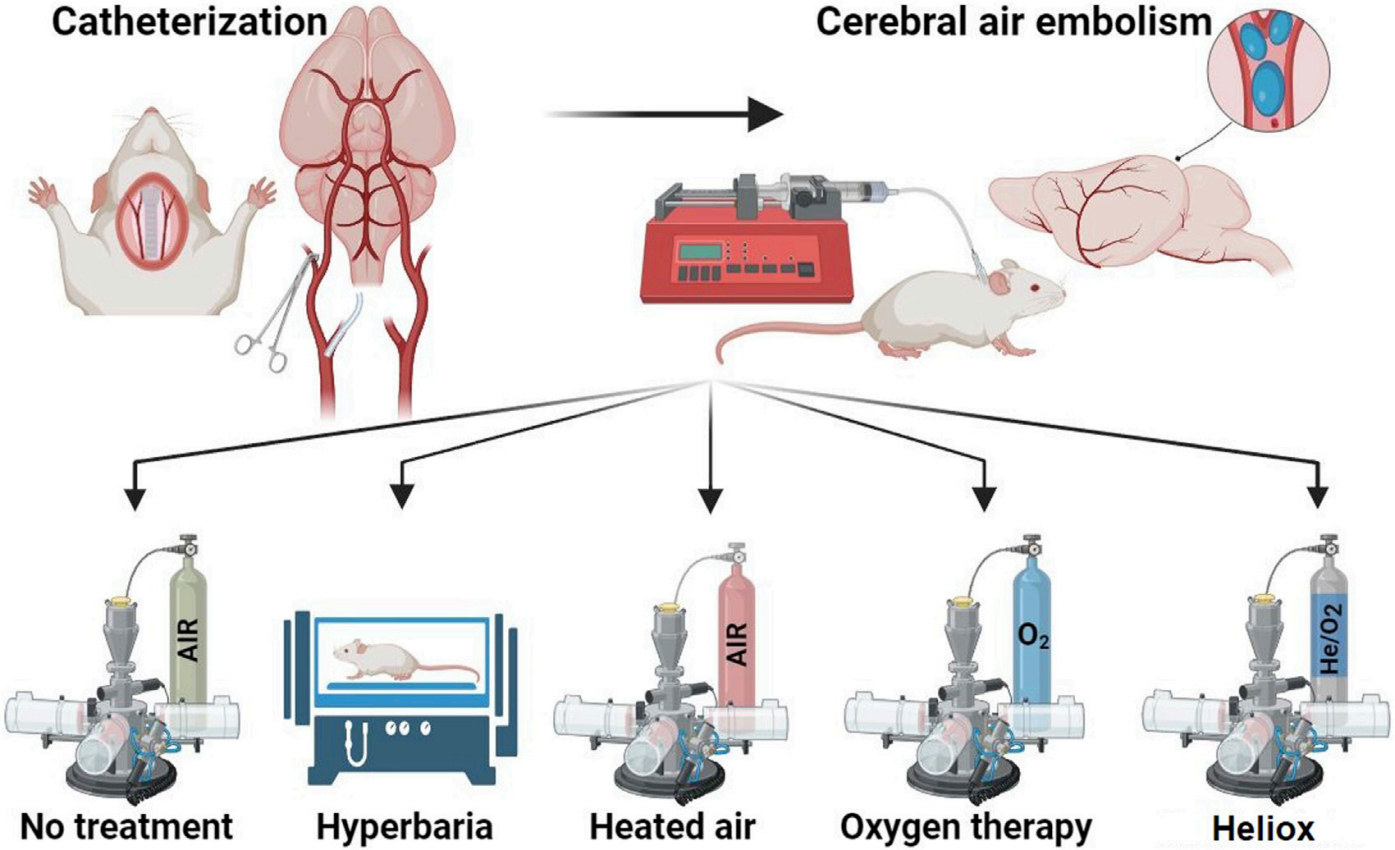
Study design. Created with BioRender.com.

Animals without any signs of clinical abnormalities were selected for the experiment. They were then distributed into five groups to ensure that the average body weight of animals at the beginning of the study did not differ between the groups. Modeling of CAE was performed on all animals. Depending on the treatment after CAE modeling, animals were divided into the following groups, eight animals each (Figure 1):

Group 1 - CAE modeling, animals were not subjected to any therapy after CAE
Group 2 - After CAE modeling, animals were given hyperbaric therapy for 30 min.
Group 3 - Animals breathed 100% oxygen for 30 min at room temperature immediately after CAE modeling. This therapy is a standard treatment for normobaric oxygen inhalation after ischemia modeling. Oxygen is used as an antioxidant agent in this case to prevent the effects of embolism (Sukhotnik et al., 2009).
Group 4 - Animals breathed heated air for 30 min with a temperature above 45°C ± 5°C immediately after CAE modeling. This group was introduced to exclude the heating factor of the inhalation mixture.
Group 5 - Animals breathed heliox for 30 min with a temperature above 45°C ± 5°C immediately after CAE modeling.

### 2.2 CAE modeling

CAE modeling was carried out in all animals at 10:00-14:00. CAE modeling was performed by delivering air through a catheter implanted in the common carotid artery the day before. To do this, a section of the external carotid artery was isolated from the animals under general anesthesia (Zoletil^®^ (VIRBAC, France) 30 mg/kg/Xyla^®^ (Interchemie werken De Adelaar, B.V., Netherlands) 10 mg/kg) and two ligatures were applied. An incision was made on the vessel between the ligatures using vascular scissors. The hole in the vessel was opened using vascular forceps, and a catheter prefilled with heparin (50 units) diluted with 0.9% NaCl physiological solution (Mosfarm, Russia) was inserted. The distal ligature was released, and the catheter was pushed further along the vessel until it reached the common trunk of the carotid artery. The catheter was then secured with a shock-absorbing ring, and the ligatures were tightened and tied. Finally, the catheter was led to the back of the neck. The external sections of the catheters measured 3.5–4.0 cm in length. The ventral wound was sutured, while the dorsal side was tightly sutured around the catheters.

The following day, CAE was modeled by connecting a 0.5 mL syringe to the catheter placed at the back of the rat’s neck, which was then placed in an automatic dispenser Aladdin AL-1000 (World Precision Instruments, INC., United States). The automatic pipette was set to deliver air at a rate of 10 μL/min. The catheter injected 100 µL of air per animal. The animals were placed in the fixation houses while awake. The syringe was filled with saline. Between the syringe and the implanted catheter there was a connecting catheter with 100 µL of air. This design made it possible to push a fixed volume of air and to control the end of the injection. We did not assess the size and number of bubbles as we focused on the total volume of air and the rate of injection.

### 2.3 Administration of gases and gas mixtures to be tested

Following the CAE modeling, the administration of tested gases and gas mixtures was carried out promptly. Inhalation of oxygen, heated air, or heated heliox was facilitated using the LIC–“Laboratory Inhalation Complex for Supplying Gas Mixtures”, developed by “Specialized Design Bureau of Experimental Equipment at the Institute of Medical and Biological Problems of the Russian Academy of Sciences”. This complex enables the heating of supplied gas mixtures. The temperature, humidity, pressure, and O2 concentration in the breathing zone of animals were monitored in real-time. The atmospheric air and heliox were heated to 45°C ± 5°C. Oxygen inhalation session was conducted with the heating radiators switched off. The animals were then placed in the LIC exposure module, which was previously filled with the gas mixture. All groups underwent continuous inhalation for 30 min.

### 2.4 Helium-oxygen mixture (heliox)

The “Specialized Design Bureau of Experimental Equipment at the Institute of Medical and Biological Problems of the Russian Academy of Sciences” produced a helium-oxygen mixture (heliox) by combining medical oxygen (with a purity of no less than 99.5%) according to GOST R 5583-78 and helium (with a purity of no less than 99.995%) according to technical certificate (TС) 0271-135–31323949-2005. The ratio of partial volumes of gases in heliox was 30% (28%–32%) oxygen and 70% (68%–72%) helium. The gas mixture’s authenticity and purity were verified at “Specialized Design Bureau of Experimental Equipment at the Institute of Medical and Biological Problems of the Russian Academy of Sciences” accredited gas testing laboratory (Register for accredited persons (RAP) record number: RA. RU.21OB46) using a specialized gas chromatograph to ensure compliance with TC 20.11.12-007–45745482-2021.

### 2.5 Medical oxygen

The main object of comparison was medical oxygen, an anti-hypoxic agent, produced by LLC “Kovrovsky Gas Plant”. The analytical passport number is 0280522/30 and the batch (series) number is 0220523.

### 2.6 Hyperbaric therapy

To confirm whether gas bubbles cause ischemic brain lesions in the CAE modeling using atmospheric air infusion into the carotid artery in awake rats, animals in group № two underwent hyperbaric therapy immediately after the CAE. The animals were placed in the experimental hyperbaric chamber “Myshka 2″, designed based on “Specialized Design Bureau of Experimental Equipment at the Institute of Medical and Biological Problems of the Russian Academy of Sciences”, and pressurized with atmospheric air up to 3 atm absolute (ATA). Pressure was monitored using a calibrated manometer MO-11202 (Manotec, Russia). The animals were maintained at this pressure for 30 min, followed by decompression. Decompression of rats was performed for 25 min after a 30-min hyperbaria exposure. The animals breathed atmospheric air under pressure.

### 2.7 Study of locomotor activity in the “open field”

Locomotor activity of the animals was recorded using a computerized system Multiple Activity Cage 47420 with CUB 2005 v.3.0.15 software (Ugo Basile, Italy). The recordings were taken before CAE modeling, 3 h and 24 h after CAE modeling, animals were tested at 10:00-14:00. The cage is equipped with infrared photosensors located around the perimeter of a transparent plastic square cage to count locomotor movements. Each animal was tested separately for a 3-min period to measure vertical and horizontal activity.

### 2.8 Respiratory parameters

Respiratory parameters, including respiratory rate and volume, were measured using the FE141 Spirometer unit on the PowerLab 8/35 computerized system (ADInstruments Pty Ltd., Australia) before CAE modeling, 3 h and 24 h after CAE modeling, animals were tested at 10:00-14:00. The animal was placed in a rat holder and given at least 2 min to calm down and assume a comfortable pose. A breathing mask was positioned over the animal’s nose and respiratory parameters were recorded for 10 s using LabChart software. The parameters were recorded three times and the averages of the three measurements were calculated. The respiratory rate (breaths per minute) and volume (in mL) were measured and used to calculate the minute respiratory volume (in mL per minute) using the formula:

Minute respiratory volume=Respiratory rate×Respiratory volume

### 2.9 Measurement of cardiovascular parameters

Systolic blood pressure and heart rate were measured before CAE modeling, 3 h and 24 h after CAE modeling, at 10:00-14:00, using a computerized NIBP PowerLab 8/35 system (ADInstruments Pty Ltd., Australia). The animals were placed in a rat holder of appropriate size with a thermostatically controlled heating pad underneath to improve blood circulation in the tail and facilitate signal transmission. The sensor was adjusted to obtain a clear pulse signal. The pulse sensor (clip) was placed on the ventral surface at the base of the tail, just below the caudal artery, to achieve a clear pulse signal. Once the animal was calm and the signal was clearly visible, a compressor was activated to inflate the cuff. The measurement was performed three times for each time point.

### 2.10 Body temperature

Rectal body temperature was measured using a WT-03 digital thermometer (B.Well, Switzerland) with a measurement range of 32°C–42°C, all animals were tested at 10:00-14:00.

### 2.11 Visualization of the damaged brain area

Twenty-four hours after CAE modeling, the animals were anesthetized with a mixture of Zoletil^®^ (VIRBAC, France) at a dose of 30 mg/kg and Xyla^®^ (Interchemie werken De Adelaar, B.V., Netherlands) at a dose of 10 mg/kg. After the onset of the surgical stage of anesthesia, the animals underwent necropsy. Necropsy involved total blood sampling from the inferior vena cava, opening of the dorsal skull vault without damaging the brain tissue, and extraction of the brain with examination of the cerebral membranes. The brain was extracted and sliced into 2 mm thick frontal sections. These sections were then placed in a 1% solution of 2,3,5-Triphenyltetrazolium chloride (TTC) (Merck, United States) and stained at 37°C for 10–12 min. Photographs of all stained sections were analyzed using ImageJ software (United States) to calculate the area of the ischemic lesions in the right cerebral hemisphere.

### 2.12 Statistical analysis

Descriptive statistics were applied to all quantitative data. Mean and standard deviation were calculated and presented in figures. Functional test data were analyzed using the nonparametric Mann-Whitney test. Brain sections data were evaluated using Fisher’s exact criterion. The statistical analysis was performed using Prism five software (GraphPad, United States). Differences were determined at *p* < 0.05.

## 3 Results

### 3.1 Locomotor activity

**FIGURE 2:**
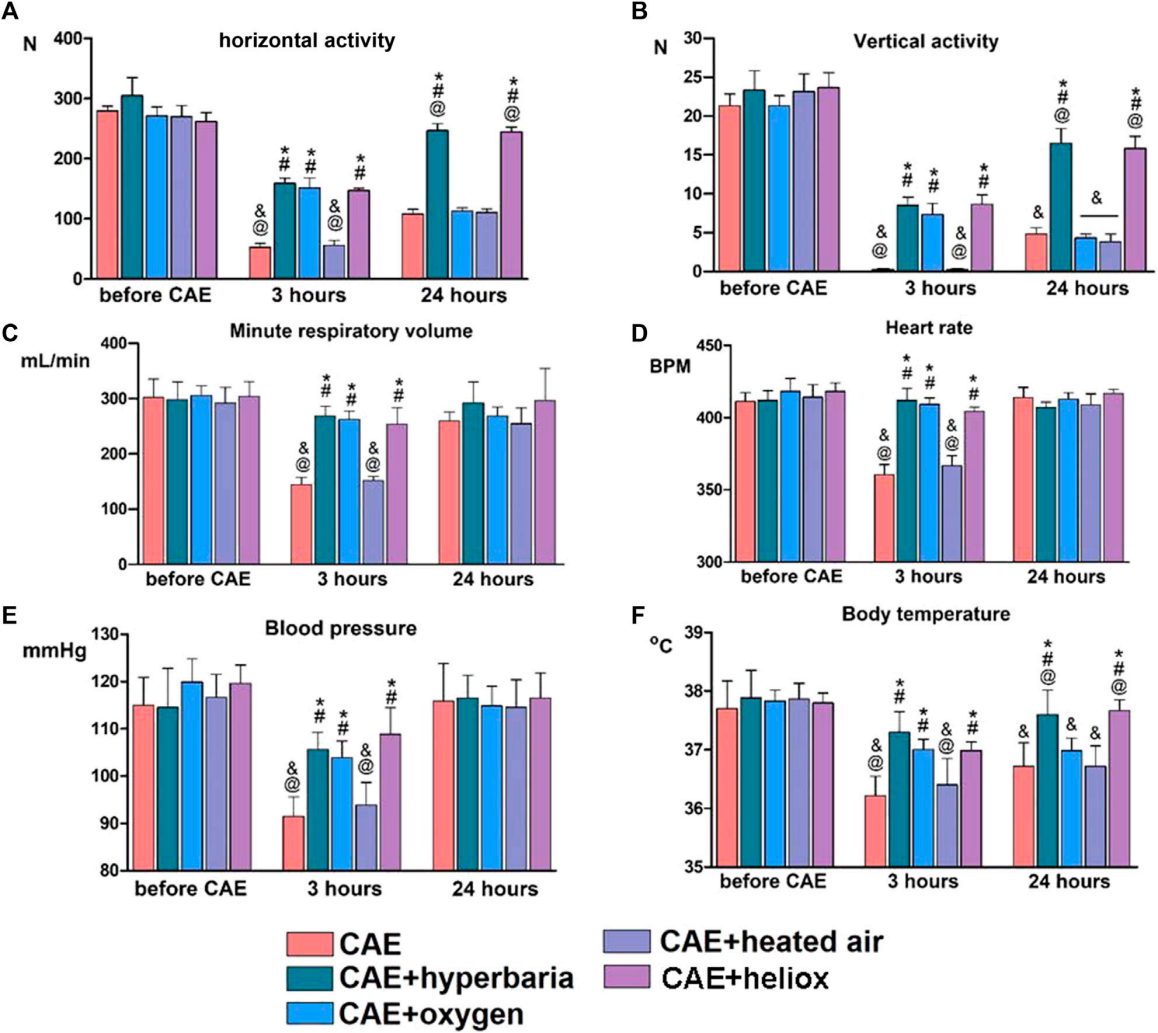
Changes in physiological parameters before CAE modeling and 3 and 24 h after CAE modeling. Vertical **(A)** and horizontal **(B)** activity of animals in the “open field”; minute respiratory volume **(C)**; heart rate **(D)** and blood pressure **(E)**; body temperature **(F)**. Statistically significant differences in Mann-Whitney test: * - *p* < 0.05 compared to CAE group; # - *p* < 0.05 compared to CAE + heated air group; @ - *p* < 0.05 compared to CAE + oxygen group; & - *p* < 0.05 compared to CAE + hyperbaria group.

Statistically significant differences in the parameters of locomotor activity were observed when animals were tested 3 and 24 h after CAE modeling (Figures 2A, B). Horizontal and vertical activity was significantly reduced 3 h after CAE modeling in the group without therapy and in group No. 4 where heated air was applied. However, the treatment with medical oxygen, hyperbaria, and heliox for 30 min had a positive effect on vertical and horizontal activity 3 h after CAE modeling. 24 h after CAE modeling, reliable recovery of vertical and horizontal activity was only observed in the groups that received hyperbaric therapy and heliox inhalation. Inhalations of oxygen and heated air did not result in any improvement of the animals’ activity.

### 3.2 Respiratory parameters

Minute respiratory volume decreased significantly after 3 h, and recovered 24 h after CAE modeling in the group without therapy (Figure 2C). In group 4, the application of heated air did not lead to the recovery of minute respiratory volume 3 h after CAE modeling. On the other hand, hyperbaric therapy, oxygen inhalation, and heliox inhalation prevented the decrease in minute respiratory volume 3 h after CAE modeling. After 24 h of CAE modeling, respiratory system parameters returned to intact level in all groups.

### 3.3 Cardiovascular parameters

Three hours after CAE modeling, animals without therapy exhibited a significant decrease in systolic blood pressure and heart rate. However, cardiovascular parameters returned to their initial levels 24 h after CAE modeling (Figures 2D, E). The use of heated air did not result in the recovery of blood pressure and heart rate 3 h after CAE modeling. However, the application of hyperbaric therapy, medical oxygen and heliox for 30 min immediately after the CAE had a significant positive effect on cardiovascular parameters. After 24 h of CAE modeling, cardiovascular parameters returned to their initial levels in all groups.

### 3.4 Body temperature

Animals without any therapy experienced decreases in body temperature 3 and 24 h after CAE modeling (Figure 2F). Additionally, inhalation with heated air did not result in the recovery of body temperature during CAE modeling. However, animals from the groups receiving hyperbaric therapy, heliox or medical oxygen had statistically significantly higher body temperatures than the group without therapy when tested 3 h after CAE modeling. Twenty-4 hours after CAE modeling, the group using heliox and hyperbaric therapy returned to baseline body temperature, while the groups treated with oxygen and heated air still had significantly lower body temperatures.

### 3.5 Assessment of ischemic brain damage

**FIGURE 3:**
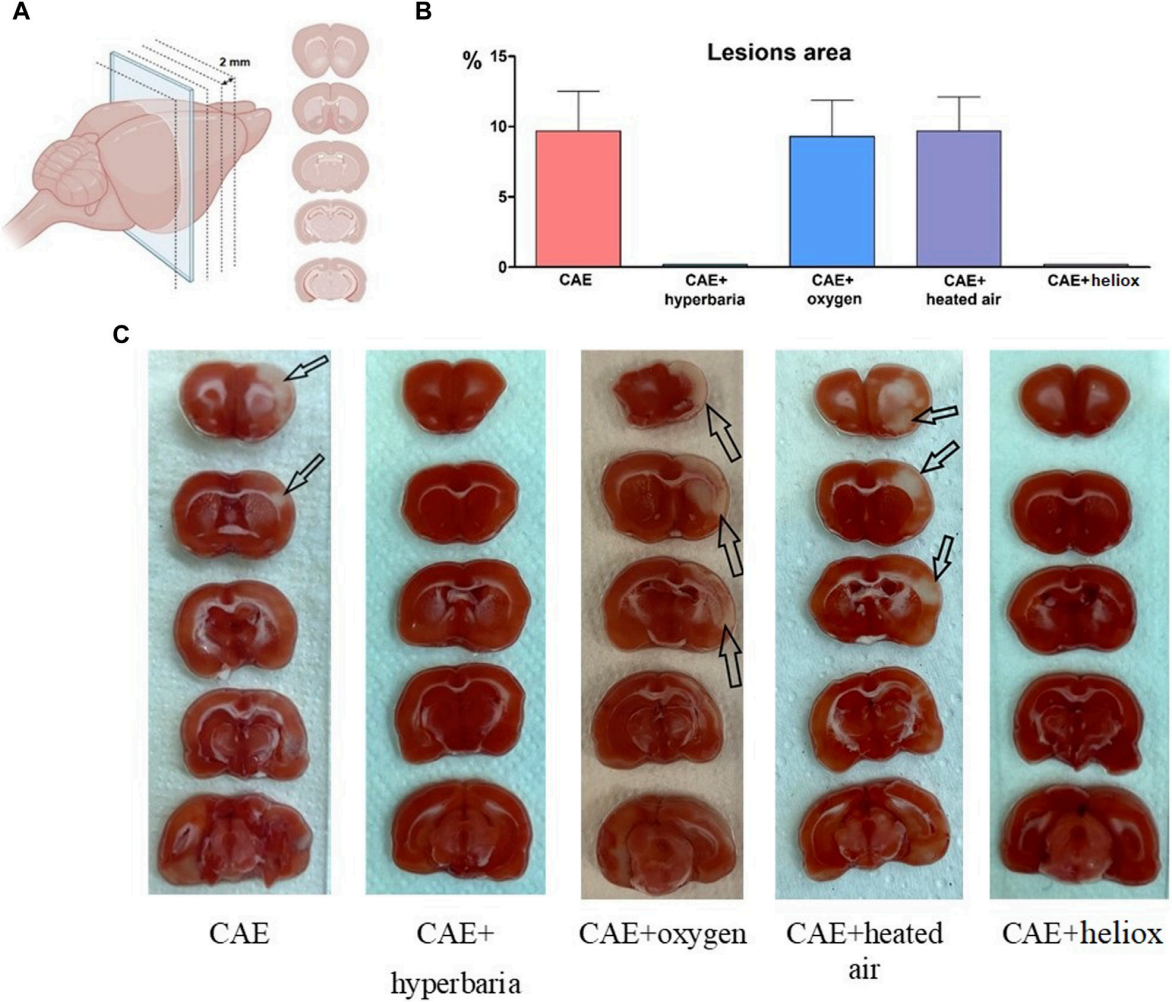
Brain slices, created with BioRender.com **(A)**; Percentage of right hemisphere lesions area 24 h after CAE modeling **(B)**; macroscopic brain slices stained with TTC 24 h after CAE modeling, arrows indicate foci of ischemic brain damage **(C)**.

Upon examining brain slices stained with TTC, we observed clearly expressed foci of ischemic brain damage in each of the animals with CAE modeling without therapy (Table 1; Figure 3). The affected area averaged 9.7% of the right hemisphere. In contrast, no lesions were detected in animals that received hyperbaric therapy immediately after CAE modeling. Therefore, brain tissue damage during CAE modeling is caused by ischemia resulting from arterial occlusion by exogenous air bubble consisting of 78% nitrogen.

**TABLE 1:** Number of ischemic brain lesions 24 h after CAE modeling.

Groups	1–CAE	2–CAE + hyperbaria	3–CAE + oxygen	4–CAE + heated air	5–CAE + heliox
Presence of lesion foci on brain slices	8/8	0/8*	8/8	8/8	0/8*

*p < 0,05 compared to group 1 (Fisher’s exact test).

Each of the animals using inhalation of medical oxygen and heated atmospheric air (groups No. 3 and No. 4) exhibited foci of ischemic brain damage, with an average area of 9.3% and 9.7% relative to the area of the right hemisphere. No visible foci of ischemia were found in the animals from group No. 5, which received inhalation with heliox immediately after CAE modeling.

## 4 Discussion

Intravascular gas embolism can be iatrogenic and can occur due to trauma or during emergency ascent from the water while diving (Kerrigan and Cooper, 2022). The immediate use of hyperbaric therapy and oxygen inhalation to enhance tissue oxygenation is the classical scheme for preventing the development of CAE. However, hyperbaric chambers can only be placed in specially prepared facilities, which must meet strict requirements, and the procedure should be performed by trained personnel (GOST R51316-99, 1999). Oxygen inhalation can also lead to toxic effects on the central nervous system due to the increased formation of reactive oxygen species, so it should be applied in batches (Heyboer et al., 2017).

We put forward the idea of using heated heliox as an alternative to emergency oxygen therapy under normobaric conditions. Previous studies have shown that heliox (70% helium/30% oxygen) is more effective than conventional oxygen therapy in correcting acute respiratory failure (Oei et al., 2010). Heliox has been found to reduce infarct volume in an animal model of focal cerebral ischemia-reperfusion (Pan et al., 2007) and cellular damage in an *in vitro* model of brain injury (Coburn et al., 2008). Furthermore, the combination of helium with oxygen has been shown to reduce the toxic effects of oxygen (Ryan et al., 2023). Heliox has been reported to have neuroprotective activity in a neonatal hypoxic-ischemic encephalopathy model (Zhong et al., 2023). Inhalation of heliox after modeling ischemic stroke by middle cerebral artery occlusion resulted in a significant reduction in neurological deficit compared to inhalation with 100% oxygen (Pan et al., 2011).

We conducted a study in which we evaluated the efficacy of heliox inhalation (30% oxygen and 70% helium) in comparison to inhalation of medical oxygen and heated air on the development of CAE. As the aim of the study was to investigate heliox as an emergency treatment for CAE, we did not use a group of animals receiving HBOT. Currently, the main emergency treatment is the inhalation of normobaric oxygen as the most accessible measure in places far from hyperbaric chambers.

Three hours after CAE modeling, animals without any therapy exhibited reduced locomotor activity, respiratory volume, respiratory rate, blood pressure, and body temperature. At necropsy, 24 h after CAE modeling, animals without therapy exhibited foci of ischemic brain damage, as well as a subsequent decrease in body temperature and locomotor activity. The study showed that modeling CAE in awake rats using atmospheric air infusion into the carotid artery causes the foci of ischemic brain damage, which occupy on average 9.7% of the area of the right hemisphere.

To confirm that gas bubbles are the cause of foci of ischemic brain damage, CAE modeling was performed using atmospheric air infusion into the carotid artery in awake rats, immediately thereafter hyperbaria was performed. Divers are known to undergo therapeutic recompression up to 3 ATA pressure (Jones et al., 2023). Therefore, we decided to place the animals at a pressure of 3 ATA to be sure that our hyperbaric therapy would certainly work. We prioritized Boyle’s physical laws and Henry’s law, which speak to the ability of gases to compress and dissolve into liquids under overpressure. Twenty-4 hours after CAE modeling and hyperbaric therapy, the animals showed no abnormalities in behavior or physiological parameters, and no ischemic brain lesions was observed. Therefore, brain tissue damage during CAE modeling is caused by ischemia resulting from occlusion of arteries by exogenous air bubbles containing 78% nitrogen (Durant and Oppenheimer, 1949). In addition to physical laws, the positive effect of hyperbaria can be explained by the short time from the onset of ischemia to recanalization of the vessel, since tissue hypoxia has not had time to develop. The hyperbaric group shows that the excess atmospheric pressure acting on the air embolus is the basis of HBOT in the first minutes after embolism. We do not exclude the possibility that similar hyperbaric therapy without oxygen may not be effective at later times.

Medical gaseous oxygen is classified as a clinical and pharmacological drug with antihypoxic properties for inhalation. It can be used to treat cerebral vascular diseases that cause tissue hypoxia. A study conducted on rats showed that normobaric oxygen therapy after middle cerebral artery occlusion/reperfusion resulted in the recovery of neurobehavioral parameters and a reduction in the area of brain lesion foci (Wang et al., 2021). Patients who underwent endovascular recanalization and received normobaric oxygen inhalation as part of their rehabilitation showed improved functional recovery and a reduced risk of ischemic brain damage, according to Cheng et al. (2021). The use of 100% oxygen in CAE may have a combined effect: to reduce the size of emboli due to the removal of nitrogen, the main component of emboli, from the vessels and, in addition, to exhibit an antihypoxic effect. Similarly, the results of the present study on animal functional activity support the feasibility of continuous medical oxygen application for 30 min after CAE modeling. However, the improvement in the rats’ wellbeing was only short-term and symptomatic. Thus, the use of oxygen will reliably reduce the clinical manifestations of CAE. But we did not see that oxygen therapy completely eliminates the effects of CAE, as hyperbaric therapy does. After 24 h of undergoing CAE, all animals showed foci of ischemic brain damage, decreased body temperature, and locomotor activity, despite receiving oxygen therapy. It is possible that oxygen reduces the area of brain lesions, but we only assessed the presence or absence of lesion foci with TTC staining, so we did not see the effects of oxygen therapy on the brain slices. Oxygen inhalation after CAE modeling briefly alleviated the development of ischemic stroke symptoms.

24 h after CAE, no brain lesion foci were found in animals that received continuous 30-min inhalation of heliox. Functional tests, including evaluation of locomotor activity, respiratory and cardiovascular systems, and body temperature measurement, showed that the use of heliox immediately after CAE modeling largely prevents the functional changes associated with brain damage. It should be noted that animals from all groups were tested at the same time (10:00-14:00). Animals from different groups were tested in a randomized order to minimize the risk of false results related to the rats’ circadian rhythms. Although rats have inactive (lights on) phase of the circadian cycle at this time, researchers test animals during daytime with lights on as well (De la Casa et al., 2018). We believe that conducting functional tests during inactive phase did not affect the interpretation of the results (Yoshizawa et al., 2019).

The application of heated heliox immediately after CAE modeling eliminates the behavioral disturbances and ischemic brain damage that occur during CAE. The damaging effects of CAE were also eliminated with the use of heated heliox, which proceeded in a similar manner to the application of hyperbaria. If hyperbaria eliminates the damaging effect of CAE by removing intravascular air bubbles, it is possible that heated heliox also removes intravascular gas bubbles, which are the cause of ischemic brain damage. The more pronounced efficacy of heliox after CAE modeling compared to oxygen is likely related to the faster rate of bubble disappearance from vessels (Hyldegaard and Madsen, 2007).

At first glance, oxygen inhalation and heliox should have a common mechanism of action, based on the concentration gradient formation between the blood and alveoli, thus accelerating the flushing of nitrogen from the blood through the lungs, which leads to the removal of emboli from the vascular bed. However, the efficacy of oxygen in our study is inferior to the therapeutic effect of heliox in CAE.

One explanation is that heliox interferes with the biochemical pathway that prevents cell death (Pagel et al., 2007; Shuai et al., 2024). This is in the phosphatidylinositol 3-kinase pathway, which leads to the activation of protein kinase B (Akt), which in turn phosphorylates GSK-3b (glycogen synthase kinase-3b) and BAD (Bcl-2-associated death promoter). Phosphorylation of GSK-3b and BAD by Akt can suppress caspase-3 activity and thereby inhibit cellular apoptosis (Datta et al., 1999). This pathway is possible because the lubricating film around the bubble, leaves room for some blood cells to continue moving through the bloodstream. The presence of space between the embolus and the vessel, allowing for prolonged microperfusion (Marsh et al., 2023). We hypothesize that helium, which is present in the blood in a gaseous state, may diffuse along a concentration gradient into the air embolus, causing an imbalance of gases within the embolus. This theory has not yet been proven. However, the comparable efficacy of hyperbaria and heliox suggests that they are acting on the same target, the air embolus.

We excluded the role of the heliox heating factor. The group that used heated air inhalation immediately after CAE modeling did not differ from the group that had CAE without therapy in functional tests and assessment of brain lesion foci.

Therefore, the use of heated heliox continuously for 30 min immediately after CAE modeling improves the physical condition of animals and prevents ischemic brain lesions.

Inhalation of heated heliox immediately after embolism protects against damage and can be clinically used both in cases of direct entry of air embolus into the bloodstream and after any injury to the alveolar-capillary membrane of the lung. The use of therapy as soon as possible after embolism is an important step in influencing the course of the disease. We have demonstrated the positive effect of heliox as an emergency, immediate treatment as an alternative to classical oxygen therapy under normobaric conditions. However, assuming that heliox acts directly on the air embolus, the use of heliox at delayed intervals, such as 3 h or more, may result in reperfusion syndrome, which is known to have greater consequences than ischemia itself.

## 5 Conclusion

Heliox (30% oxygen and 70% helium) improves the physiological state of animals and prevents the formation of ischemic brain lesions when it is applied immediately after CAE modeling in awake rats by continuous inhalation with a heated heliox for 30 min on a par with hyperbaric therapy. In turn, oxygen inhalation briefly normalized some physiological parameters, but had no effect on the brain lesion foci. The use of heated atmospheric air immediately after CAE modeling had no effect on the course of the disease. Thus, inhalation of heated heliox may be an effective and safe alternative for emergency care in situations with a risk of CAE.

## Data Availability

The raw data supporting the conclusion of this article will be made available by the authors, without undue reservation.
